# Identification of a novel *MEF2C::SS18L1* fusion in childhood acute B-lymphoblastic leukemia

**DOI:** 10.1007/s00432-024-05846-8

**Published:** 2024-06-22

**Authors:** Chuqin Chen, Jiali Wang, Meiyun Kang, Peng Wu, Liwen Zhu, Yongjun Fang, Yao Xue

**Affiliations:** 1 Department of Hematology and Oncology, Children’s Hospital of Nanjing Medical University, 72# Guangzhou Road, Nanjing, 210008 Jiangsu Province China; 2 Key Laboratory of Hematology, Nanjing Medical University, Nanjing, China

**Keywords:** B-acute lymphoblastic leukemia, Fusion gene, MEF2C::SS18L1, MEF2D::SS18, MEF2C::SS18

## Abstract

**Purpose:**

Leukemia-associated fusion genes are closely related to the occurrence, development, diagnosis, and treatment of leukemia. DNA microarrays and second-generation sequencing have discovered multiple B-ALL fusion genes. We identified a novel *MEF2C::SS18L1* fusion gene in a child diagnosed with B-ALL. This study investigates the oncogenicity and prognosis of this fusion gene in B-ALL.

**Methods:**

A child with B-ALL who has a *MEF2C::SS18L1* fusion is reported as a newly discovered case. Compared the breakpoints, structural domains, clinical phenotypes, and differential expression genes of *MEF2C::SS18L1* and *MEF2D::SS18.*Using “ONCOFUSE” software, the carcinogenicity of *MEF2C::SS18L1* is predicted. Using whole transcriptome sequencing, we analyze the breakpoints and the secondary structure of the fusion protein. Further, we compared the structures, differentially expressed genes, and clinical phenotypes of *MEF2D* and *MEF2C* fusion genes by DESeq, GO functional enrichment, and flow cytometry immunophenotyping analysis.

**Results:**

Whole transcriptome sequencing identified a *MEF2C::SS18L1* fusion transcript in a 3-year-old child with B-ALL. The MADS box, MEF structural domain, HJURP_C structural domain, and TAD I structural domain of *MEF2C*, and the QPGY structural domain of *SS18L1*, make up the fusion protein. “Oncofuse” found a 0.99 Bayesian probability that the fusion gene drives cancer. The breakpoint positions, fusion protein secondary structures, differentially expressed genes, and clinical characteristics of this patient were identical to those with *MEF2D::SS18* fusion gene.

**Conclusion:**

We identified a novel *MEF2C::SS18L1* fusion gene in childhood ALL, which shares similar structural and clinical characteristics with *MEF2D::SS18*. Further studies with more samples should be conducted in future.

**Supplementary Information:**

The online version contains supplementary material available at 10.1007/s00432-024-05846-8.

## Introduction

Acute lymphocytic leukemia (ALL), a common malignancy in children, often arises from interactions between exogenous (e.g., environmental exposure) or endogenous (e.g., genetic susceptibility) factors. Usually, genetic mutations may lead to transformation of lymphoid progenitor cells (Kuiper et al. 2007). According to the immunophenotype of tumor cells, ALL is divided into acute B-lymphocytic leukemia (B-cell precursor acute lymphoblastic leukemia, B-ALL) and acute T-lymphocytic leukemia (T-cell precursor acute lymphoblastic leukemia, T-ALL) (Arber et al. 2016).

About 75% of B-ALL patients exhibit abnormal chromosomal numbers or translocations (Mullighan 2012), which can generate fusion genes, including *ETV6::RUNX1, TCF3::PBX1, BCR::ABL1, MLL*-related fusion genes, etc. (Harrison and Foroni 2002; Onciu 2009). There are growing evidence suggesting that gene fusion is initial event in oncogenesis (Mitelman et al. 2007) and plays an important role in cases of aggressive cancer (Villanueva 2012). Early fusion interferes with the expression of hematopoietic-related genes and oncogenes, contributing to the development of B-ALL. In recent years, many new fusion genes have been detected in the development and relapse of B-ALL by DNA microarray and second-generation sequencing. These fusion genes are mainly involved in B-cell developmental processes such as cell cycle, apoptosis, proliferation, autophagy, and epigenetic regulation (Collins-Underwood and Mullighan 2010; Mullighan et al. 2007; Forero-Castro et al. 2016; Zakaria et al. 2017).

In our study, we describe a case with *MEF2C::SS18L1*, a novel fusion gene unprecedentedly detected in a 3-year-old boy diagnosed with B-ALL. We analyzed the oncogenicity of *MEF2C::SS18L1* and its association with the prognosis of B-ALL. Our study sheds new light on the possible pathogenesis of B-ALL associated with *MEF2C (myocyte enhancer factor 2C)* fusion.

## Materials and methods

### Case

A 3-year-old child with primary B-ALL was included. Transcriptome analysis confirmed the presence of the *MEF2C::SS18L1* fusion. Peripheral blood (PB) and bone marrow (BM) samples were collected from the patient for diagnosis and medical analysis. This study was approved by the Ethics Committee of the Children’s Hospital of Nanjing Medical University. The patient’s parents provided written informed consent to participate in this study.

### Karyotyping and fluorescence in situ hybridization (FISH)

Conventional karyotyping was performed after short-term culture, and every 20 metaphase cells after G-binding were analyzed. Karyotypes were described according to the International System for Human Cytogenetic Nomenclature (ISCN 2016). FISH was carried out on every 500 interphase cells using the Vysis LSI JAK2 dual-color break-apart probe (Abbott Laboratories) according to the manufacturer’s recommendations.

### Flow cytometry (FCM) immunophenotyping and fusion gene detection

Heparin-anticoagulated BM samples were used for the immunophenotyping. For each tube, at least 3 × 10^5^ leucocytes were stained with the following monoclonal antibodies: *CD34, CD117, CD10, CD19, CD20, CD79a, CD2, CD4, CD8, CD3, CD7, CD5, CD13, CD33, CD14, CD64, CD11b, HLA-DR, MPO*, and *CD45*. Then, 2 × 10^4^ target cells were obtained by the FACS Canto Plus flow cytometer (BD Biosciences). The immunophenotypes of abnormal juvenile cells were analyzed using the FACSDiva software (BD Biosciences). BM leucocytes were enriched using ACK lysis buffer, and total RNA was extracted with Trizol. Then 500 ng of RNA was transcripted into cDNA by random primers and Moloney Murine Leukemia Virus Reverse Transcriptase (Progema, Beijing). Based on qRT-PCR, a multi-fusion gene detection system, and the 43 Fusion Gene Screening Kit (Yuanqi Biopharmaceutical, Shanghai, China), was used to screen transcripts.

### RNA sequencing (RNA-Seq) and fusion validation

Ribosomal RNA was removed from the total RNA by the Ribozero method and then subjected to cDNA synthesis. cDNA was used as a template to construct the library for sequencing. Whole messenger transcriptome sequencing was performed on the Illumina Hiseq X sequencing platform. Sequenced fragments were aligned with the UCSC hg19 reference genome by STAR software. FusionCatcher was used for gene fusion prediction. The downstream genes of the fusion gene were analyzed by variant effector prediction (VEP). The fusion gene was annotated in databases including Clinvar, dbSNP, 1000genome, genomeAD, ExAC, COSMIC, etc. RNA-Seq results were validated by RT-PCR, followed by Sanger sequencing.

### Oncofuse to predict oncogenic potential

Oncofuse (http://www.unav.es/genetica/oncofuse.html) is employed to predict the oncogenic potential of fusion genes found by Next-Generation Sequencing in cancer cells. It is a post-processing step to validate in silico the predictions made by fusion detection software.The pipeline was executed by simply running a Java or Groovy script with some parameters on a standardized input file (all required packages were installed automatically via Groovy or Grape). The parameters are set based on features present in known oncogenic fusions. A complete list of features was shown in paper of Shugay et al. (Shugay et al. 2013). We provided IDs of fusion gene partners as well as locations of breakpoints (intron/exon ID and coordinate) within the major Refseq transcript of each gene.

### Gene expression analysis

The quality of FastQ data for this patient was assessed by FastQC and controlled by Trim-Galore. Then the reads were mapped to the GRCh38 reference genome by Hisat2. The dataset GSE11504 was downloaded from the GEO, and gene expression data in children and adult bone marrows were collected. The dataset GSE11504 contained 25 cases of healthy children, adolescents and adults aged 2 months to 28 years. Since the gene expression in this dataset was detected by microarray, the batch effect was removed by the SVA package.

### DEGs and enrichment analyses

The DEGs were calculated by the DESeq2 package according to | log2-fold-change |> 5 and adj. P-value < 1e-10. To pinpoint the DEGs induced by *MEF2C::SS18L1* fusion, we constructed a protein–protein interaction network based on the STRING database, then filtered out the DEGs connected to *MEF2C* or *SS18L1* for further analysis. The biological function of these DEGs was evaluated with GO analysis from the clusterProfiler package.

## Results

### Case presentation

**Fig. 1:**
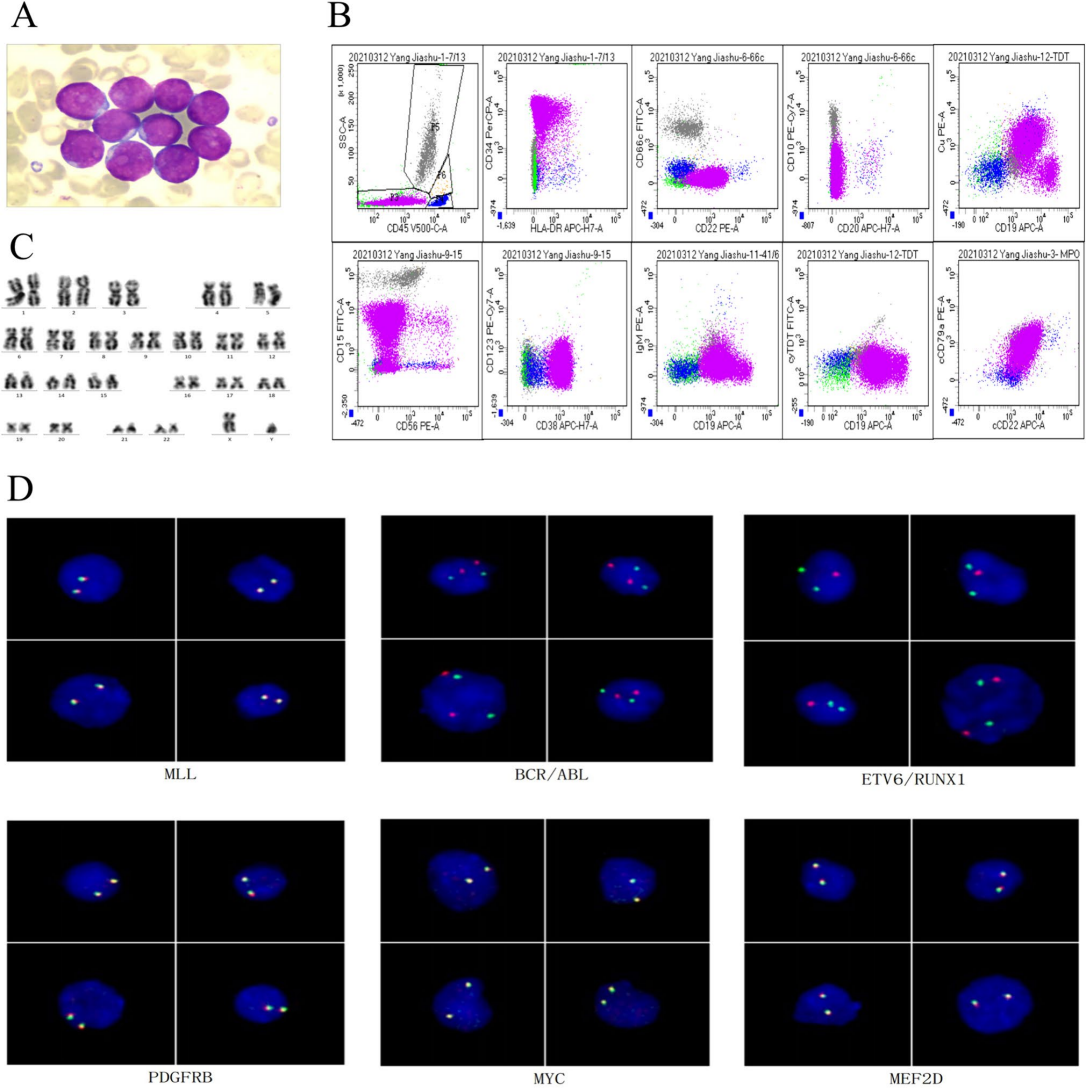
Morphology, karyotyping, FISH analysis, and Immunophenotype. **A** Bone marrow (BM) smear at admission; **B** Immunophenotyping of primary BM samples by FCM; **C** G-band karyotype of the BM sample at admission; **D** Representative interphase nuclei in the primary BM sample

This 3-year-old boy had suffered intermittent joint pain for about a month, which was self-relieving. Two weeks later, he was admitted to the hospital for persistent pain in the left hand joint. Blood testing showed a white blood cell count of 8.97 × 10^9^/L, a hemoglobin level of 101 g/L, and a platelet count of 332 × 10^9^/L. Morphologic examination of BM smears disclosed markedly active BM and lymphatic proliferation, with 93.0% of lymphocytes being primitive and juvenile (Fig. 1A). FCM revealed that about 81.0% of B lymphocytes in the BM were abnormally juvenile and positive for *CD19*, *cCD79a*, *cCD22*, and *CD22* (Fig. 1B). The chromosomal karyotype was normal (Fig. 1C). FISH analysis showed negative results about *MLL* rearrangement, *BCR/ABL* fusion, *ETV6/RUNX1* fusion, *PDGFRB* isolation, *MYC* disruption, and *MEF2D* disruption (Fig. 1D). Multiplex-nested RT-PCR, designed to amplify 43 fusion transcripts, was negative (Supplement Table 1). The copy number variation (CNV) assay did not detect large fragment deletions or duplications in *IKZF1*. *MEF2C::SS18L1* fusion transcripts and *PTPN11* mutation were detected by next-generation whole transcriptome sequencing. The child was diagnosed with B-ALL, initially stratified as low-risk, and treated with the CCCG-ALL2020 regimen with no significant adverse effects. MRD on day 19 was 12.41%, and the percentage of juvenile lymphocytes was 4.0%. Due to his poor response to the initial treatment, the risk was elevated to intermediate, according to CCCG ALL 2020. He was then given CAT chemotherapy, with MRD < 0.01% and the *MEF2C::SS18L1* fusion gene turning negative on day 46, as well as a complete remission eventually achieved. The child is now on sequential chemotherapy.

**Table 1:** Predicted oncogenicity of MEF2C-SS18L1

Genomic coordinates	5’FPG	3’FPG	P-value	Probability of being a “driver”
chr5:88,044,886 > chr20:60,737,808	*MEF2C*	*SS18L1*	0.01	0.99

### Identification of *MEF2C::SS18L1* fusion transcript by RNA-Seq

**Fig. 2:**
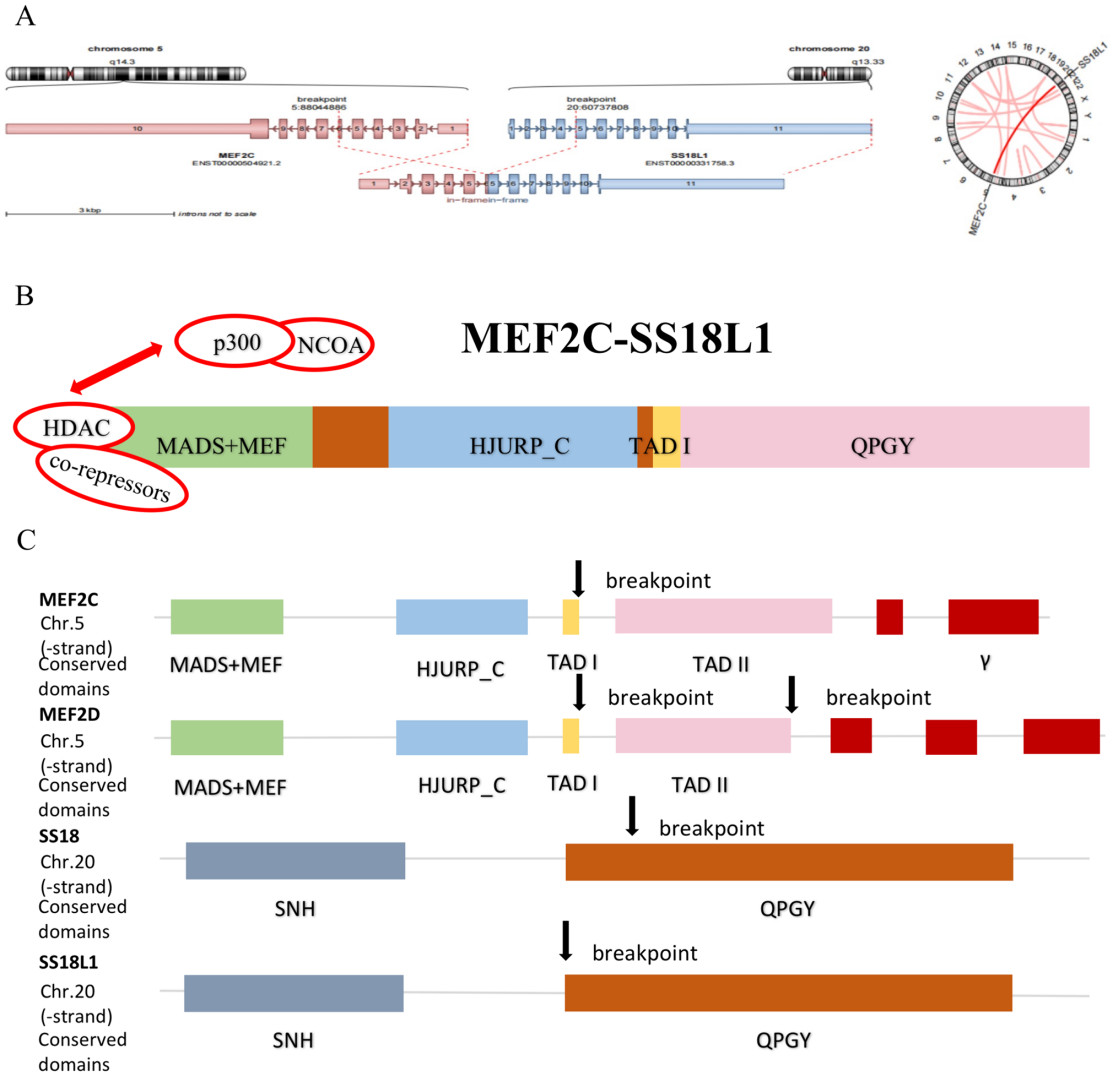
*MEF2C::SS18L1* fusion and comparison of its secondary structures with *MEF2D::SS18* fusion protein. **A** RNA-Seq revealed breakpoints in exon 6 of *MEF2C* and exon 5 of *SS18L1*; **B** Schematic diagram of predicted secondary structure of *MEF2C::SS18L1* fusion protein; **C** Comparison of *MEF2C::SS18L1* and *MEF2D::SS18* breakpoints

The primary BM sample was analyzed by RNA-Seq. We identified a novel *MEF2C::SS18L1* fusion in B-ALL. RNA-Seq results indicated that the breakpoints were located in exon 6 of *MEF2C* on chromosome 5 and exon 5 of *SS18L1* on chromosome 10 (Fig. 2A). The fusion protein consisted of a MADS box and a MEF domain, a HJURP_C structural domain, a TAD I structural domain in *MEF2C*, and the QPGY structural domain in *SS18L1* (Fig. 2B).

### Predicted oncogenicity of *MEF2C::SS18L1*

We entered the breakpoint information of the *MEF2C::SS18L1* fusion gene into "Oncofuse", and discovered that the fusion gene had a Bayesian probability of 0.99 of acting as an oncogenic driver (P < 0.05). The prediction results also demonstrate protein domains, respectively, retained in the 5’ fusion partner gene and the 3’ fusion partner gene (Table 1; Fig. 2B).

### DEGs and GO annotations of *MEF2C::SS18L1*

**Fig. 3:**
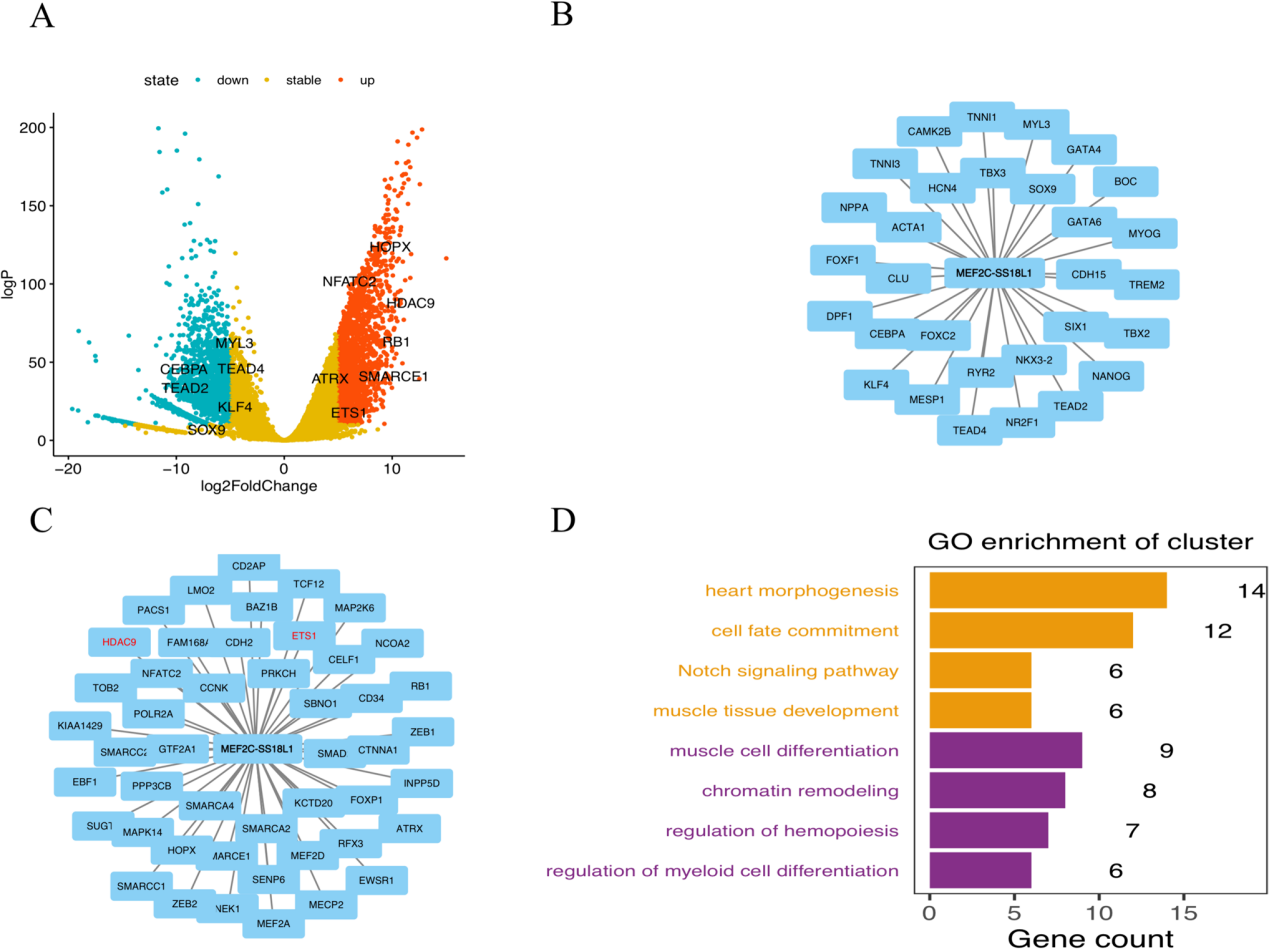
DEGs and GO annotations of *MEF2C::SS18L1.* **A** Top 13 most significant DEGs; **B** 32 down-regulated genes associated with *MEF2C* or *SS18L1* in the PPI network; **C** 49 up-regulated genes associated with *MEF2C* or *SS18L1* in the PPI network; **D** GO annotations for the biological function of the DEGs

The DEGs between normal and B-ALL tissues were screened. We obtained 1782 up-regulated and 2429 down-regulated genes using the DESeq2 package, with thresholds of | log2-fold-change |> 5 and adj. P-value < 1e-10. The top 13 most significant DEGs (*HOPX, NFATC2, HDAC9, RB1, SMARCE1, ATRX, ETS1, MYL3, TEAD4, CEBPA, TEAD2, KLF4,* and *SOX9*) were shown on the volcano map (Fig. 3A). In order to obtain the DEGs induced by the *MEF2C::SS18L1* fusion, we identified 81 DEGs associated with *MEF2C* or *SS18L1* through the STRING database, including 32 down-regulated and 49 up-regulated, as shown in PPI maps (Fig. 3B-C). In GO enrichment analysis, these DEGs were associated with heart morphogenesis (GO:0003007), cell fate commitment (GO:0045165), Notch signaling pathway (GO:0007219), muscle tissue development (GO:0060537), muscle cell differentiation (GO:0042692), chromatin remodeling (GO:0006338), regulation of hemopoiesis (GO:1,903,706), and regulation of myeloid cell differentiation (GO:0045637) (Fig. 3D).

## Discussion

In this study, we identified a novel *MEF2C::SS18L1* fusion gene associated with childhood B-ALL. After 19 days of treatment, this three-year-old patient’s MRD was still 12.4%, and the percentage of juvenile lymphocytes was 4.0%. Response to early treatment (induction therapy) remains the most reliable independent factor for predicting the prognosis of childhood ALL, which is assessed internationally based on the prednisone sensitivity test (Conter et al. 2010). MRD is the strongest predictor for the long-term survival of ALL patients (Schultz et al. 2007). A retrospective analysis found that D19 MRD ≥ 1% during the early induction phase of chemotherapy was an independent risk factor for poor prognosis (Yu et al. 2020). The high MRD in the present case suggests that the new fusion gene may affect patients’ responses to treatments. Thus, we further analyzed the fusion gene with “Oncofuse”, finding that *MEF2C::SS18L1* is a "driver" of ALL.

Currently, *MEF2C::SS18* fusion has been reported in microsecretory adenocarcinoma (MSA), a novel subtype of salivary gland adenocarcinoma that tends to be less malignant or appears as an inert salivary gland tumor (Bishop et al. 2019). MSA has characteristic histologic and immunophenotypic features and most of MSA patients have *MEF2C::SS18* fusion, which was identified as a typical marker in this disease. In a clinical study conducted by Justin A. Bishop et al., a total of 24 MSA cases were collected, and *MEF2C::SS18* fusion was confirmed in 21 of them. The tumors exhibited consistent histologic features including: (1) microcystic ducts, (2) flattened intercalated duct-like cells, (3) monochromatic oval hyperpigmented nuclei, (4) abundant basophilic luminal secretions, (5) fibromuscular-like stroma, and (6) subtle infiltration of the periphery. These tumors were uniformly positive for S100 (24 of 24) and p63 (24 of 24) (Bishop et al. 2021). *SS18 (SS18 subunit of BAF chromatin remodeling complex)* is a gene with similar structure of *SS18L1 (SS18L1 subunit of BAF chromatin remodeling complex)*. An analysis of *SS18* and *SS18L1* sequences has revealed that both proteins contain an SS18 N-terminal (SNH) domain and a QPGY domain (Kato et al. 2002). Therefore, it is reasonable to infer that there is similarity between *MEF2C::SS18* and *MEF2C::SS18L1*. Although there is not any reports of *MEF2C::SS18L1* in hematologic tumors at present, the presence of recurrent *MEF2C::SS18* fusions in MSA suggests the importance and specificity of this fusion in pathogenesis of malignant tumor. Notably, all of these 24 MSA cases exhibited S100 and P63 positivity, which has also been reported to be associated with the development and prognosis of hematologic neoplasms. In a retrospective cohort study, a team of researchers evaluated the levels of inflammatory markers such as S100 protein in 128 children with pre-B ALL. They concluded that S100 could be used as a biomarker to assess ALL prognosis (Brix et al. 2023). Meanwhile, P63, as a member of the P53 family, shows different expression and function in different types and stages of leukemia (Xie and Xie 2013; Pruneri et al. 2005). However, whether S100 and P63 positivity are equally present in children with *MEF2C::SS18L1* positive ALL requires further validation.

What role does the *MEF2C::SS18L1* play in ALL? Growing evidence suggests that *MEF2C* is essential for the normal hematopoietic system, particularly the production of immature and mature lymph-like cells (Schüler et al. 2008). Integrated *MEF2C* and ectopic *MEF2C* expression are found in 20% of patients with acute myeloid leukemia (AML) (Schwieger et al. 2009). In a study based on gene expression data from 117 patients with incipient T-ALL, a new subpopulation named pre-T-cell (ETP) ALL has been identified, featuring early T-cell developmental arrest and various chromosomal rearrangements leading to constitutive activation of *MEF2C* (Homminga et al. 2011). The above implies that ectopic expression of *MEF2C* is involved in the development of T-ALL and AML. Strikingly, no *MEF2C* aberrations in B-ALL have been documented in previous studies. *MEF2C* is abundantly expressed in both hematopoietic stem cells (HSC) and common myeloid progenitor cells (CMPs). *MEF2C* expression gradually decreases during the maturation of granulocyte-monocyte progenitors (GMPs) and megakaryocyte-erythroid progenitors. Compared with that in HSCs and CMPs, *MEF2C* expression is higher in common lymphoid precursors (CLPs), and decreases when the cells commit to the B-cell lineage. In contrast, *MEF2C* expression is virtually absent in T cells (Canté-Barrett et al. 2014). So, it is suggested that in normal development, *MEF2C* helps to drive differentiation into the CLP lineage or B-cell lineage. This lineage direction may be due to active transcription as *MEF2C* cooperates with p300/CBP to acetylate histones (Canté-Barrett et al. 2014).

A genomic study by Gu et al. identified fusions between *MEF2D (myocyte enhancer factor 2D)* and five genes (*BCL9, CSF1R, DAZAP1, HNRNPUL1,* and *SS18*) in 22 B-ALL cases (Gu et al. 2016). Among them, *MEF2D::SS18* fusion has caught our eye. *MEF2D* and *MEF2C* belong to the *MEF2* protein family, which consists of four members: *MEF2A, B, C,* and* D*. *MEF2* family members have multiple splicing variants and share a conserved N-terminal MADS box and MEF structural domain (Black and Olson 1998). MADS box and MEF structural domain regulate the transcriptional activity of *MEF2* by recruiting co-activators or co-repressor factors (Black and Olson 1998). As mentioned above, *SS18* and *SS18L1* sequences are similar and both of them has a QPGY domain (Kato et al. 2002). The QPGY domain is essential for transcriptional activation, while the SNH domain acts as an interaction interface for a plethora of proteins, several of which are involved in epigenetic gene regulation, including *SWI/SNF* proteins (Bruijn and Geurts van Kessel 2006). By comparing the breakpoints and structural domains of *MEF2C::SS18L1* and *MEF2D::SS18* fusion genes, we found that they retained the MADS box and MEF structural domain in MEF2 and the QPGY domain in *SS18* (Fig. 2C). *SS18* exerts its regulatory role through protein–protein interactions, but both fusion genes have lost their SNH domains; thus, we speculate that they might have lost the functions of *SS18*. Gu et al. found that all *MEF2D* fusion partners can augment *MEF2D* transcriptional activation (Gu et al. 2016). We notice a γ region in some *MEF2C* isoforms that functions to suppress the transcriptional activity of *MEF2C* and is spliced out in many tissues due to a unique 3’-splice acceptor site in *MEF2C*. The activity of the g domain is repressed by phosphorylation of serine 396 (S396), thus facilitating sumoylation at lysine 391 (K391) of *MEF2C* and the recruitment of unknown co-repressors to inhibit transcription (Kang et al. 2006). However, the *MEF2C::SS18L1* fusion gene has lost the exon that encodes the γ region, so we infer that *MEF2C::SS18L1* fusion might also enhance *MEF2C* expression.

It has been reported that *MEF2D* rearrangements can enhance its transcriptional activity and lymphoid transformation, thus contributing to the development of a high-risk leukemia (Gu et al. 2016; Yasuda et al. 2016). Fusion with *MEF2D* can significantly up-regulate *HDAC9* and *HDAC11*, activate the *MAPK* pathway, inhibit the expression of B-cell differentiation-related genes, and hinder V(D)J rearrangement, thereby blocking B-cell differentiation and maturation (Zhang and Meng 2022). *HDAC11* competes with *P300* in binding to *MEF2*, and *p300/CBP* can affect lineage direction (Fig. 2B). The immunophenotype of *MEF2D*-rearranged ALL is characterized by weak or absent expression of *CD10* and overexpression of *CD38* antigens (26). However, low or absent expression of *CD10* is a feature of *MLL*-rearranged ALL. Both the 5-year event-free survival (EFS) and overall survival (OS) rates are significantly lower in patients with *MEF2D* fusion than in other ALL patients, indicating that *MEF2D* fusion is significantly associated with ALL prognosis (Gu et al. 2016; Zhang and Meng 2022; Ohki et al. 2019). Although their response to steroids is sensitive, *MEF2D* fusion patients still show a significantly worse prognosis, with more than half experiencing relapse or dying within 1 year (Ohki et al. 2019).

Since these two fusion proteins have structurally similar domains, would *MEF2C::SS18L1*-positive B-ALL patients have similar clinical, pathological, or genetic features as *MEF2D::SS18* positive patients? In our study, we found that this patient with *MEF2C::SS18L1* fusion also showed: (1) high expression of *HDAC9,* (2) *deletion of CD10* and (3) high expression of *CD38.* Coincidentally, this is consistent with the performance of *MEF2D::SS18* fusion positive ALL. However, further in-depth studies are needed to determine whether these two fusion genes have the same pathogenic mechanism. In addition, whole transcriptome sequencing revealed a mutation in the *PTPN11* gene in this patient. A mutational analysis of RNAseq data showed that this mutation was also found in *MEF2D* fusion-positive patients (Gu et al. 2016). The co-occurrence of *PTPN11* mutation and fusion gene indicated similar molecular mechanism of *MEF2D* fusion with our present *MEF2C* gene fusion. However, further molecular experiments are still needed.

There are some limitations of the present study. First, we have searched public databases and have not found whole transcriptome sequencing data from the bone marrow of healthy subjcts. Therefore, in the present DEGs analysis, we used dataset GSE11504 as control set, which is microarray data contained 25 cases of healthy children, adolescents and adults aged 2 months to 28 years. This microarray contains 47,000 transcripts, representing 38,500 human genes. Expression information of this dataset can be widely used for the discovery of new regulatory pathways, exploration of disease mechanisms, and discovery of biomarkers (Vitari et al. 2011). We also used SVA package to remove the batch effect. Second, as *MEF2C::SS18L1* fusion gene is a novel identified fusion gene in the present ALL case, limited clinical expression and clinical data could be obtained. Currently we have only one clinical sample, and *MEF2C::SS18* fusion has only been reported in Microsecretory Adenocarcinoma without public expression data. We will continue to follow more ALL patients with *MEF2C::SS18L1* fusion gene to perform more analyses on gene expression, as well as their clinical outcomes.

In summary, we identified a new *MEF2C::SS18L1* fusion gene in a child with B-ALL that has similar structure and clinical features to *MEF2D::SS18*. Unlike those with *MEF2D* fusion, this patient showed high expression of the *EST1* gene. This patient has achieved complete remission and is on sequential chemotherapy. We will continue to follow him to further observe his prognosis. We suggest that physicians should re-evaluate the risk once *MEF2C* fusions are present in B-ALL. Meanwhile, we should further explore the mechanism of this fusion gene and develop targeted drugs to improve the prognosis of ALL patients.

### Supplementary Information

Below is the link to the electronic supplementary material.

Supplementary file1 (DOC 217 KB)

## Data Availability

The patient’s data are available upon reasonable request.
