# Supplementary material for: Identification of a novel MEF2C::SS18L1 fusion in childhood acute B-lymphoblastic leukemia
Source: J Cancer Res Clin Oncol. 2024 Jun 22;150(6):314. doi: 10.1007/s00432-024-05846-8 (PMC11193691; doi:10.1007/s00432-024-05846-8)
Supplement: Supplementary file 1 — Supplementary file1 (DOC 217 KB) [file 432_2024_5846_MOESM1_ESM.doc]

**Supplement table 1** Leukemia 43 fusion gene detection kit (Shanghai Yuanqi Biotechnology Co., Ltd.)

| Reaction Fluid No. | Name of the fusion gene detected |
| --- | --- |
| PCR reaction solution 1 | BCR-ABL |
|  | SIL-TAL1 |
|  | E2A-HLF |
| PCR reaction solution 2 | TEL-AML1 |
|  | MLL-AF4 |
|  | E2A-PBX1 |
| PCR reaction solution 3 | AML1-ETO |
|  | MLL-AF9 |
|  | PML-RARα |
| PCR reaction solution 4 | PLZF-RARα、STAT5b-RARα |
|  | MLL-(AF6、AF10、ELL、ENL) |
|  | NPM-MLF1 |
| PCR reaction solution 5 | TEL-PDGFRB |
|  | FIP1L1-PDGFRA |
|  | AML1-MDS1/EVI1、AML1-MTG16 |
| PCR reaction solution 6 | CBFβ-MYH11 |
|  | DEK-CAN |
|  | Internal reference |
| PCR reaction solution 7 | TEL-ABL |
|  | ETV6-PDGFRA |
|  | NUP98-(HoxA13、HoxC11、HoxD13、HoxA9、HoxA11、PMX1) |
| PCR reaction solution 8 | TEL-JAK2 |
|  | MLL-(AF17、AF1q、AF1p、AFX、SEPT6) |
|  | (NPM、FIP1L1、PRKAR1A、NUMA1)- RARα |
| PCR reaction solution 9 | NPM-ALK |
|  | SET-CAN |
|  | TLS-ERG |
